# Predictors of remission with etanercept-methotrexate induction therapy and loss of remission with etanercept maintenance, reduction, or withdrawal in moderately active rheumatoid arthritis: results of the PRESERVE trial

**DOI:** 10.1186/s13075-017-1484-9

**Published:** 2018-01-16

**Authors:** Josef S. Smolen, Annette Szumski, Andrew S. Koenig, Thomas V. Jones, Lisa Marshall

**Affiliations:** 1 Division of Rheumatology, Department of Medicine 3, Medical University of Vienna, Waehringer Guertel 18-20, 1090 Vienna, Austria; 2 Hietzing Hospital, Vienna, Austria; 3 inVentiv Health, Princeton, NJ USA; 4 Pfizer, Collegeville, PA USA

**Keywords:** Rheumatoid arthritis, Treatment, Remission, Low disease activity, Etanercept, Methotrexate

## Abstract

**Background:**

The aim was to analyze characteristics that predict remission induction and subsequent loss of remission in patients with moderately active rheumatoid arthritis (RA) who received full-dose combination etanercept plus methotrexate induction therapy followed by reduced-dose etanercept or etanercept withdrawal.

**Methods:**

Patients with Disease Activity Score based on 28-joint count (DAS28) >3.2 and ≤5.1 received open-label etanercept 50 mg once weekly (QW) plus methotrexate for 36 weeks. Those who achieved DAS28 low disease activity by 36 weeks were randomized to double-blind treatment with etanercept 50 mg or 25 mg QW plus methotrexate or placebo plus methotrexate for 52 weeks. All analyses were adjusted for the continuous baseline variables of their respective remission outcomes.

**Results:**

Younger age, body mass index (BMI) <30 kg/m^2^, and lower Health Assessment Questionnaire (HAQ) score at baseline were significant predictors of week-36 remission (*P* < 0.05) based on DAS28, Simplified Disease Activity Index (SDAI), and Clinical Disease Activity Index (CDAI). Baseline DAS28, SDAI, and CDAI were significantly predictive of all three remission endpoints (*P* < 0.05). For all three treatments, the strongest predictors of loss of DAS28 remission included failure to achieve sustained remission (DAS28 < 2.6 at weeks 12, 20, 28, and 36) with induction therapy, higher DAS28/SDAI/CDAI at randomization and at 1 month, increase in DAS28/SDAI/CDAI at 1 month, and increase in DAS28/CDAI/SDAI components and patient-reported outcomes (PROs) at 1 month. With the exception of not achieving sustained remission, very similar significant predictors were observed for loss of SDAI and CDAI remission.

**Conclusion:**

These findings suggest that patients with moderately active RA who are younger and have lower BMI, lower HAQ, and lower disease activity at baseline are most likely to achieve remission when receiving combination etanercept and methotrexate induction therapy. In addition, patients who fail to achieve sustained remission with induction therapy and those with worse disease activity and PROs at early time points after initiating maintenance therapy with a full-dose or reduced-dose etanercept-methotrexate regimen or methotrexate monotherapy are most likely to lose remission across all treatment arms. These findings may help guide clinicians’ decision-making as they treat patients to remission and beyond.

**Trial registration:**

ClinicalTrials.gov, NCT00565409. Registered on 28 November 2007

**Electronic supplementary material:**

The online version of this article (10.1186/s13075-017-1484-9) contains supplementary material, which is available to authorized users.

## Background

Clinical remission is a critical treatment target in all patients with rheumatoid arthritis (RA), regardless of the level of their disease activity [1]. In the clinical practice setting, a large proportion of patients have moderately active RA [2, 3], which is associated with an elevated risk of joint destruction and functional disability [4, 5]. Although treatment with biologics such as tumor necrosis factor (TNF) inhibitors is effective in inducing and maintaining remission in patients with RA, the determining factors associated with remission induction with biologic therapy, and remission maintenance after such therapy is reduced or discontinued, have not yet been well-studied. However, this information would be important to clinicians and patients when they are deciding on a treatment plan to limit costs and patient exposure.

The PRESERVE trial was an induction/maintenance study of the biologic etanercept in which investigators assessed the consequences of etanercept dose reduction or withdrawal in adults with moderately active RA who achieved low disease activity (LDA) after 36 weeks of treatment with full-dose etanercept combined with methotrexate. The conventional full-dose or reduced-dose etanercept-methotrexate combination regimens were shown to be more effective in maintaining LDA than methotrexate alone after etanercept withdrawal [6]. Post hoc analyses of findings from the PRESERVE trial were conducted here to identify potential predictive markers for remission induction and loss after modification of etanercept dosing.

## Methods

The methodology of the PRESERVE trial has been described in detail in a previous publication [6] and is briefly summarized in the following sections. In the last decade, a Disease Activity Score in 28 joints (DAS28, on the basis of erythrocyte sedimentation rate (ESR)) <2.6 was commonly considered to indicate remission in practice and clinical trials, including the PRESERVE trial [6, 7]. When the PRESERVE trial was designed and the statistical analyses planned, the American College of Rheumatology/European League Against Rheumatism (ACR/EULAR) Boolean definition of remission had not yet been published [8]. Although DAS28 < 2.6 no longer defines remission according to ACR/EULAR criteria, as it better represents minimal disease activity than remission [8], and other cut points for remission have been proposed [9–11], in these analyses, remission continues to be defined as DAS28 < 2.6 in agreement with the primary manuscript. However, ACR/EULAR index-based remission criteria were also evaluated.

### Study design

All patients enrolled in the initial open-label period were treated with etanercept 50 mg once weekly (QW) plus methotrexate for 36 weeks. The dose of methotrexate at screening was continued in the open-label period, but dose adjustments were permitted at the investigator’s discretion. Patients who achieved sustained LDA, defined as an average DAS28 ≤ 3.2 from weeks 12 to 36 and DAS28 ≤ 3.2 at week 36, after 36 weeks of combination etanercept-methotrexate treatment were randomized to continue weekly subcutaneous injections of etanercept 50 mg, reduce the etanercept dose to 25 mg, or withdraw etanercept and receive placebo injections, all in addition to background methotrexate, for the subsequent 52-week double-blind period. In the double-blind period, the same dose of methotrexate was administered as in the last 8 weeks of the open-label period.

### Patients

Adults who were enrolled in this study had active RA with moderate disease activity (DAS28 > 3.2 and ≤5.1) at screening and baseline and had received stable methotrexate doses. Randomized patients who received double-blind treatment had completed the first 36 study weeks and achieved sustained LDA.

### Post hoc analyses

The statistical analysis plans for the exploratory/post hoc predictor analyses are available in Additional file 1: Appendices 1 and 2.

#### Predictors of remission induction

Randomized patients were included in analyses of baseline variables; patients who were dosed and had week-36 visit data were included in post-baseline analyses. Patients who had week-36 DAS28/Simplified Disease Activity Index (SDAI)/Clinical Disease Activity Index (CDAI) values were included in the respective post hoc analysis of predictors of DAS28/SDAI/CDAI remission in the open-label period. Remission in this analysis was defined as DAS28 < 2.6, SDAI ≤3.3, or CDAI ≤2.8 after 36 weeks of full-dose combination therapy. Demographic characteristics and baseline disease characteristics—including clinical, patient-reported, and radiographic measures—were examined in the post hoc analyses as possible predictors of remission. Univariate logistic regression of each remission endpoint was conducted on each baseline predictor, treated as continuous (i.e., age, body mass index (BMI), and disease duration; anti-citrullinated peptide antibody (ACPA) and rheumatoid factor (RF) levels; C-reactive protein (CRP) and ESR levels; DAS28, SDAI, and CDAI; physician and patient global assessments (PGA and PtGA); Health Assessment Questionnaire (HAQ) score; and the van der Heijde modified total Sharp score (mTSS) and erosion and joint space narrowing (JSN) scores), or as categorical (i.e., age >40 vs. ≤40, >40 to ≤65 vs. ≤40, and ≥65 vs. <65 years; sex, male vs. female; BMI, 18.5–25 vs. <18.5 kg/m^2^, 25–30 vs. <18.5 kg/m^2^, ≥30 vs. <18.5 kg/m^2^; disease duration, ≤6 vs. >24 months, >6 to ≤12 vs. ≤6 months, >12 to ≤24 vs. ≤6 months; ACPA and RF status, positive vs. negative; CRP and ESR, > vs. ≤ upper limit of normal (ULN); DAS28, 4.1–4.4 vs. ≤4.1, 4.4–4.7 vs. ≤4.1, and >4.7 vs. ≤4.1; SDAI, 15.5–18.4 vs. ≤15.5, 18.4–21.8 vs. ≤15.5, and >21.8 vs. ≤15.5; CDAI, 14.5–17 vs. ≤14.5, 17–20 vs. ≤14.5, and >20 vs. ≤14.5; and HAQ score, >0.5 to ≤1.0 vs. ≤0.5, >1.0 to ≤1.5 vs. ≤0.5, and >1.5 vs. ≤0.5; and prior/current smoker vs. non-smoker), and adjusted for the respective baseline DAS28, SDAI, or CDAI.

Stepwise logistic regression was performed on the following predictors selected by the authors based on their clinical relevance: age, ≤40, >40 to ≤65, and >65 years; BMI, <18.5 vs. ≥18.5 to <25 vs. ≥25 to <30 vs. ≥30; baseline CRP, ≤ULN vs. >ULN to 3*ULN vs. >3*ULN; sex, male and female; smoking status, prior/current vs. non-smoker; disease duration, ≤6, >6 to ≤12, >12 to ≤24, and >24 months; and HAQ score, ≤0.5, >0.5 to ≤1.0, >1.0 to ≤1.5, and >1.5. In addition, although radiographic variables were included in univariate analyses, they were excluded from the stepwise analyses because week-36 x-ray data were not available for approximately 65 patients.

#### Predictors of loss of remission

Patients in the modified intent-to-treat (mITT) population of the randomized treatment period (i.e., patients who received at least one dose of study drug and had at least one DAS28 evaluation in the randomized treatment period) who had achieved DAS28 remission (DAS28 < 2.6) at week 36 were examined to identify predictors of loss of DAS28 remission in this period. (As previously mentioned, more stringent definitions of remission have been proposed [8, 12]; however, the DAS28 < 2.6 cut point was used to define remission in the original PRESERVE trial and was therefore also applied in this publication for consistency.) Demographic and disease characteristics reported at baseline of the open-label period (week 0) and included in this predictor analysis were age, sex, BMI, disease duration, and ACPA and RF status. Clinical, functional, and radiographic outcomes observed at baseline of the randomized treatment period (week 36) and analyzed as week-36 predictors were DAS28 and change in DAS28 from baseline to week 36; tender joint count (TJC) and swollen joint count (SJC); CRP and ESR levels; PGA and PtGA; general health and pain scores (visual analog scale); HAQ score; and the mTSS, erosion, and JSN scores. With the exception of radiographic variables (mTSS, erosion, and JSN), which were not collected at week 40, all of these predictors were also analyzed at week 40, as well as change in the predictors from week 36 to week 40. The following categories of achievement of sustained remission in the open-label period were also analyzed as predictors: non-sustained vs. sustained remission (i.e., DAS28 < 2.6 at weeks 12, 20, 28, and 36) and sustained remission by duration (i.e., sustained remission at weeks 12, 20, 28, and 36 vs. weeks 20, 28, and 36 vs. weeks 28 and 36 vs. only at week 36).

Cox proportional hazards modeling, adjusted for baseline of the respective remission endpoints, was used to determine the relationship between the first loss of DAS28, SDAI, and CDAI clinical remission and the demographic and clinical predictors. A more stringent remission endpoint, loss of DAS28 remission plus a change in DAS28 ≥ 0.6 in the double-blind period, was also analyzed. Higher hazard ratios (HRs) denoted greater potential for the loss of remission. However, each predictor has a different range of values; because HRs are based on these ranges, HRs cannot be compared. Each predictor was analyzed for each treatment arm separately and therefore HRs cannot be compared between the treatment arms. Stepwise models for each endpoint and each treatment were created to determine which subsets of predictors were the strongest. To explore the effects of clinical response to full-dose combination induction therapy on subsequent response after tapering or withdrawal of etanercept in the double-blind period, the relationships between week-36 DAS28, SDAI, and CDAI and the probability of loss of DAS28, SDAI, and CDAI remission or LDA in the double-blind period was analyzed in patients receiving reduced-dose combination therapy or methotrexate monotherapy after randomization, using univariate logistic regression models.

Descriptive summary statistics were also provided for mean DAS28 (95% confidence interval (CI)) during the double-blind period in patients who never lost remission at any time point during the period and those who did lose remission during the period, among patients who had achieved DAS28 remission at week 36.

## Results

### Patients

A detailed description of overall patient disposition in the PRESERVE study was previously published [6]; a summary of patients included in the current predictor analyses is provided in Additional file 2: Figure S1. Demographics and baseline disease characteristics of patients in the overall population in the open-label period and the randomized population of the double-blind period are shown in Table 1. At the start of the open-label period, 83% of patients were women, mean age was 48 years, and disease duration, 6.9 years; the mean DAS28, SDAI, and CDAI scores were 4.4 (standard deviation (SD), 0.4), 19.1 (SD, 5.1), and 17.8 (SD, 5.0), respectively. Disease, patient-reported, and radiographic characteristics were similar among the three treatment groups at baseline of both study periods.

**Table 1:** Demographic and disease characteristics at baseline for the overall patient population (mITT) in the open-label period and for the treatment group subpopulations (mITT) in the randomized, double-blind period [6]

	Open-label population	Randomized population					
Demographic/disease characteristics at baseline	ETN 50 mg + MTX *N* = 834	ETN 50 mg + MTX *n* = 202		ETN 25 mg + MTX *n* = 202		MTX *n* = 200	
Age, years	48.4 (11.9)	48.1 (12.0)		46.4 (12.2)		48.3 (12.2)	
Female, *n* (%)	694 (83.2)	164 (81.2)		157 (77.7)		167 (83.5)	
Prior tobacco use, *n* (%)	158 (18.9)	42 (20.8)		31 (15.4)		39 (19.5)	
Disease duration, years	6.9 (7.0)	6.8 (7.2)		6.4 (7.1)		7.3 (6.7)	
ACPA positive, *n* (%)	642 (77.6)	161 (80.1)		156 (77.6)		156 (78.8)	
RF positive, *n* (%)	603 (72.7)	147 (73.1)		142 (70.7)		147 (74.2)	
Clinical and patient-reported characteristics at baseline and randomization	ETN 50 mg + MTX *N* = 834	ETN 50 mg + MTX *n* = 201		ETN 25 mg + MTX *n* = 201		MTX *n* = 197	
	Week 0	Week 0	Week 36	Week 0	Week 36	Week 0	Week 36
DAS28	4.4 (0.4)	4.3 (0.5)	2.0 (0.6)	4.4 (0.4)	2.1 (0.6)	4.3 (0.4)	2.1 (0.6)
SDAI	19.1 (5.1)	18.7 (4.8)	4.7 (3.6)	19.2 (5.1)	4.8 (3.2)	18.8 (5.4)	4.8 (3.2)
CDAI	17.8 (5.0)	17.5 (4.6)	4.1 (3.5)	17.9 (5.0)	4.2 (3.2)	17.8 (5.3)	4.3 (3.2)
TJC, 0–28	5.1 (2.9)	4.7 (2.7)	0.6 (1.2)	5.2 (2.9)	0.7 (1.3)	5.1 (2.9)	0.7 (1.2)
SJC, 0–28	3.8 (2.6)	3.9 (2.7)	0.6 (1.5)	3.8 (2.6)	0.6 (1.2)	4.0 (2.7)	0.6 (1.1)
CRP, mg/L	12.3 (16.4)	11.9 (13.9)	5.9 (5.9)	12.8 (18.0)	6.0 (6.5)	10.4 (13.1)	5.2 (3.3)
ESR, 0–100 mm/hour	22.2 (13.1)	22.2 (12.9)	9.9 (7.2)	21.7 (13.4)	10.7 (8.6)	20.4 (12.1)	9.6 (6.0)
PGA, 0–10	4.1 (1.3)	4.0 (1.3)	1.1 (0.9)	4.0 (1.3)	1.2 (1.1)	4.2 (1.3)	1.1 (0.8)
PtGA, 0–10	4.9 (1.7)	4.9 (1.8)	1.8 (1.7)	4.8 (1.7)	1.8 (1.5)	4.6 (1.7)	1.9 (1.6)
General health VAS, 0–100 mm	43.4 (17.0)	43.2 (17.3)	14.1 (15.8)	41.5 (15.5)	14.8 (15.0)	40.9 (15.6)	15.1 (15.5)
Pain VAS, 0–100 mm	45.5 (17.4)	46.1 (17.8)	12.8 (15.5)	43.1 (16.1)	13.8 (14.8)	44.1 (16.3)	14.2 (15.6)
Total HAQ, 0–3	1.1 (0.6)	1.1 (0.6)	0.5 (0.5)	1.1 (0.6)	0.5 (0.5)	1.1 (0.6)	0.5 (0.4)
Radiographic characteristics at baseline and randomization	ETN 50 mg + MTX *N* = 709	ETN 50 mg + MTX *n* = 184		ETN 25 mg + MTX *n* = 184		MTX *n* = 167	
	Week 0	Week 0	Week 36	Week 0	Week 36	Week 0	Week 36
mTSS (0–448)	39.3 (55.3)	42.6 (58.8)	42.7 (58.8)	39.1 (60.3)	38.9 (59.8)	42.3 (47.5)	42.4 (47.6)
Erosion score (0–280)	24.8 (33.2)	25.8 (34.6)	25.8 (34.6)	24.7 (36.8)	24.7 (36.5)	26.2 (28.1)	26.1 (28.1)
JSN score (0–168)	14.5 (23.6)	16.8 (25.3)	16.9 (25.4)	14.4 (24.8)	14.2 (24.6)	16.1 (21.1)	16.1 (21.2)

*ACPA* anti-citrullinated peptide antibody, *CDAI* Clinical Disease Activity Index, *CRP* C-reactive protein, *DAS28* Disease Activity Score based on a 28-joint count, *ESR* erythrocyte sedimentation rate, *ETN* etanercept, *HAQ* health assessment questionnaire, *JSN* joint space narrowing, *mITT* modified intention-to-treat, *mTSS* modified total Sharp score, *MTX* methotrexate, *PGA* physician global assessment, *PtGA* patient global assessment, *RF* rheumatoid factor, *SDAI* Simplified Disease Activity Index, *SJC* swollen joint count, *TJC* tender joint count, *VAS* visual analog scale

### Predictors of induction of remission

**Fig. 1:**
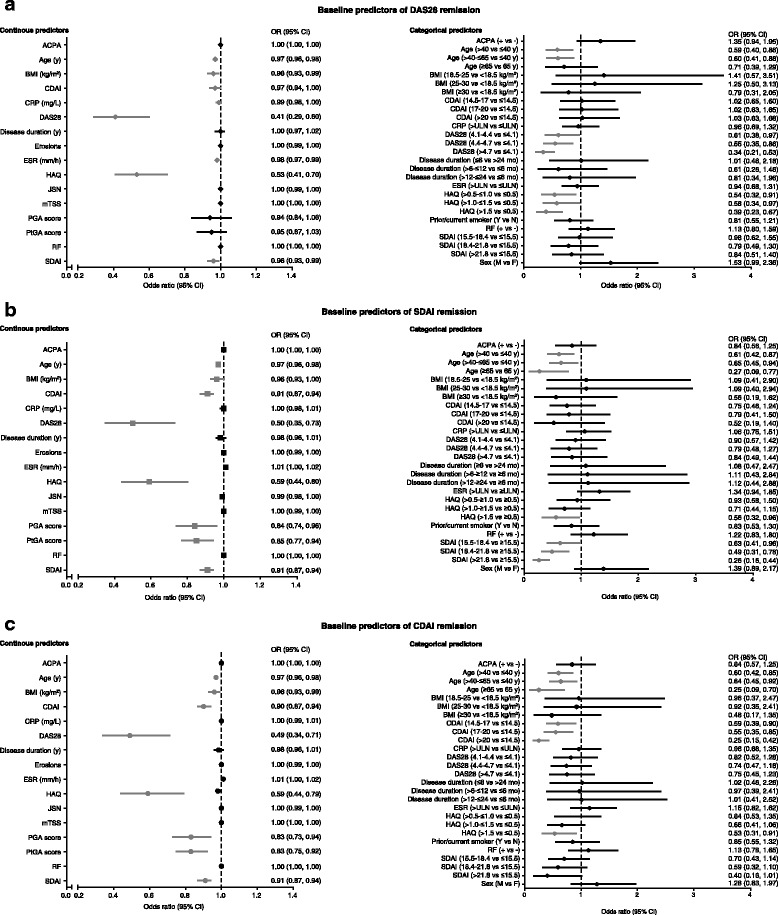
Continuous and categorical baseline predictors of disease activity score based on 28 joints (DAS28) (**a**), Simplified Disease Activity Index (SDAI) (**b**), and Clinical Disease Activity Index (CDAI) (**c**) remission using univariate logistic regression (unadjusted). If the 95% CI does not contain the value 1.0, the predictor is statistically significant at α = 0.5. Non-significant predictors, black; significant predictors, gray. For continuous predictors with odds ratios >1, higher values are associated with a greater likelihood of remission. For numeric categorical predictors with odds ratios >1, higher numeric subgroups are associated a greater likelihood of remission (e.g., patients with body mass index (BMI) 18.5–25 kg/m^2^ are more likely to achieve DAS28 remission than patients with BMI <18.5 kg/m^2^); for nominal categorical predictors, the first named subgroup is associated with a greater likelihood of remission (e.g., anti-citrullinated peptide antibody (ACPA) + patients are more likely to achieve DAS28 remission than ACPA- patients). Conversely, for continuous predictors with odds ratios <1, lower values are associated with a greater likelihood of remission. For numeric categorical predictors with odds ratios <1, lower numeric subgroups are associated with a greater likelihood of remission (e.g., patients who are ≤40 years of age are more likely to achieve DAS28 remission than patients who are >40 years of age); for nominal categorical predictors with odds ratios <1, the second named subgroup is associated with a greater likelihood of remission (e.g., patients with erythrocyte sedimentation rate (ESR) levels ≤ upper limit of normal (ULN) are more likely to achieve DAS28 remission than patients with ESR > ULN). BL*,* baseline; CI, confidence interval; CRP, C-reactive protein; HAQ, Health Assessment Questionnaire; JSN, joint space narrowing: mTSS, modified total Sharp score: PGA, physician global assessment; PtGA patient global assessment; RF, rheumatoid factor. Analyses were adjusted for baseline DAS28, SDAI, and CDAI (respectively). Patients included in the DAS28, SDAI, and CDAI remission models: n = 763, n = 755, and n = 762, respectively. Radiographic variables were excluded from the stepwise analyses because 55 patients did not have radiographic data

Based on univariate logistic regression, several continuous baseline factors were found to be predictive of DAS28, SDAI, and CDAI remission after 36 weeks of full-dose combination therapy when adjusting for their respective baseline DAS28, SDAI, and CDAI measurements (Fig. 1a–c; Table 2). Lower baseline DAS28 (unadjusted), SDAI (unadjusted), and CDAI (unadjusted), younger age (adjusted), BMI <30 kg/m^2^ (adjusted), and lower HAQ score (adjusted) were significant predictors of achievement of all three remission endpoints.

**Table 2:** Summary of (a) significant baseline factors predictive of DAS28, SDAI, and CDAI remission induction after 36 weeks of full-dose etanercept-methotrexate therapy in the open-label period and (b) significant post-baseline factors predictive of DAS28, SDAI, and CDAI remission loss at a single time point in all treatment groups in the double-blind period of the PRESERVE trial

**(a)**		
**Baseline predictors of remission induction**		
**DAS28**	**SDAI**	**CDAI**
Continuous variables		
• **Younger age**	• **Younger age**	• **Younger age**
• **Lower BMI**	• **Lower BMI**	• **Lower BMI**
• **Lower DAS28, SDAI, and CDAI**	• **Lower DAS28, SDAI, and CDAI**	• **Lower DAS28, SDAI, and CDAI**
• **Lower HAQ**	• **Lower HAQ**	• **Lower HAQ**
	• Lower PGA and PtGA	• Lower PGA and PtGA
Categorical variables		
**Younger age (>40 vs. ≤40 years;** **>40 to ≤65 vs. ≤40 years)**	**Younger age (>40 vs. ≤40 years;** **>40 to ≤65 vs. ≤40 years**; ≥65 vs. <65 years)	**Younger age (>40 vs. ≤40 years;** **>40 to ≤65 vs. ≤40 years**; ≥65 vs. <65 years)
• Lower DAS28 (≤4.1)	• Lower SDAI (≤15.5)	• Lower CDAI (≤14.5)
• **Lower HAQ** (>0.5 to ≤1.0 vs. ≤0.5; >1.0 to ≤1.5 vs. ≤0.5; **>1.5 vs. ≤0.5**)	• **Lower HAQ (>1.5 vs. ≤0.5)**	• **Lower HAQ (>1.5 vs. ≤0.5)**
**(b)**		
**Post-baseline predictors of remission loss**		
**DAS28**	**SDAI**	**CDAI**
• Lack of sustained DAS28 remission		
• DAS28 at wk 36		
• **DAS28, SDAI, and CDAI at wk 40**	• **DAS28, SDAI, and CDAI at wk 40**	• **DAS28 and CDAI at wk 40**
• **∆ in DAS28,** SDAI, **and CDAI from wk 36-40**	• **∆ in DAS28,** SDAI, **and CDAI from wk 36 to 40**	• **∆ in DAS28 and CDAI from wk 36 to 40**
• TJC and SJC at wk 40		• TJC at wk 40
• ∆ in TJC and SJC from wk 36 to 40		• ∆ in TJC from wk 36 to 40
• ESR at wk 40		
• ∆ in ESR from wk 36 to 40		
• ∆ in HAQ from wk 36 to 40		• ∆ in HAQ from wk 36-40
• **PGA, PtGA, general health VAS, and pain VAS at wk 40**	• **PGA, PtGA, general health VAS, and pain VAS at wk 40**	• **PGA, PtGA, general health VAS, and pain VAS at wk 40**
• **∆ in PGA, PtGA, general health VAS, and pain VAS from wk 36 to 40**	• **∆ in PGA, PtGA, general health VAS, and pain VAS from wk 36 to 40**	• **∆ in PGA, PtGA, general health VAS, and pain VAS from wk 36 to 40**

**Bold face** denotes predictors of remission induction and remission loss across all three sets of criteria, i.e., Disease Activity Score in 28 joints (DAS28), Simplified Disease Activity Index (SDAI), and Clinical Disease Activity Index (CDAI)

*BMI* body mass index, *HAQ* Health Assessment Questionnaire, *PGA* physician global assessment, *PtGA* patient global assessment, *TJC* tender joint count, *SJC* swollen joint count, *VAS* visual analog scale

When analyzed as continuous variables, lower DAS28, SDAI, and CDAI at baseline were significant predictors of week-36 DAS28, SDAI, and CDAI remission, respectively. Lower baseline SDAI and CDAI as continuous variables were also predictive of DAS28 remission, after adjusting for baseline DAS28. Lower baseline DAS28 and CDAI as continuous variables were predictors of SDAI remission after adjusting for baseline SDAI, and lower baseline DAS28 and SDAI as continuous variables were predictors of CDAI remission after adjusting for baseline CDAI. Lower baseline PGA and PtGA as continuous variables were significant predictors of SDAI and CDAI remission, but not DAS28 remission.

Logistic regression analyses of categorical predictors of DAS28, SDAI, and CDAI remission, shown in Fig. 1a–c, indicated that both younger age (≤40 years) and lower HAQ (≤0.5) were significant predictors for DAS28, SDAI, and CDAI remission, even after adjustment for baseline of the respective outcomes. Neither RF nor ACPA status, when analyzed as continuous predictors (i.e., RF and ACPA levels) or categories (i.e., negative or positive), was predictive of DAS28, SDAI, or CDAI remission at week 36.

**Fig. 2:**
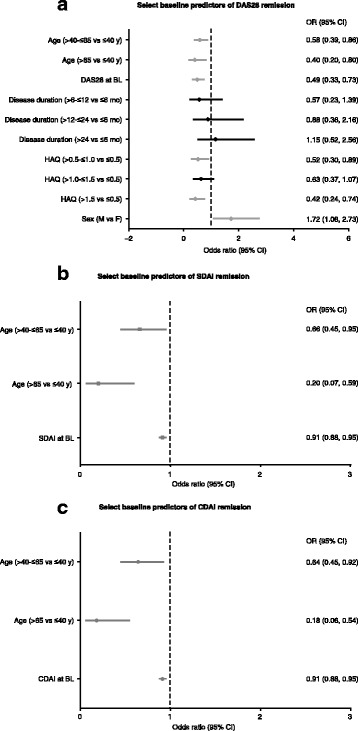
Select continuous and categorical baseline predictors of Disease Activity Score based on a 28-joint count (DAS28) (**a**), Simplified Disease Activity Index (SDAI) (**b**), and Clinical Disease Activity Index (CDAI) (**c**) remission using stepwise models (adjusted for other variables). If the 95% confidence interval (CI) does not contain the value 1.0, the predictor is statistically significant at α = 0.5. Non-significant predictors, black; significant predictors, gray. HAQ, health assessment questionnaire

In stepwise analyses of remission, including continuous and categorical analyses of baseline variables, age 40 years or younger at baseline and baseline DAS28, SDAI, and CDAI predicted achievement of the remission endpoints (Fig. 2a–c). Patients with a HAQ score ≤0.5 vs. >0.5 and who were male were significantly more likely to achieve DAS28 remission.

### Loss of remission

**Fig. 3:**
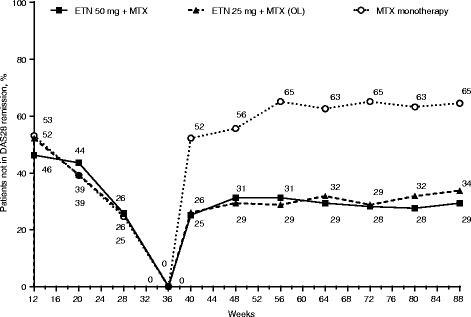
Proportions of patients who were not in Disease Activity Score based on a 28-joint count (DAS28) remission in the double-blind period. ETN, etanercept; MTX, methotrexate

At week 40 and thereafter in the double-blind period, approximately 25–34% of patients were not in DAS28 remission in the full-dose and reduced-dose combination therapy groups (Fig. 3). Although only patients who had achieved DAS28 remission were selected for the predictor analyses in the double-blind period, this rate of non-remission in the latter period was consistent with that observed at week 28 in the open-label period. At the first post-randomization visit (week 40), decreased DAS28 response was observed in all groups. In contrast, during this time frame, the proportion of patients in the etanercept withdrawal group who were not in DAS28 remission ranged from 52 to 65%. At week 88 at the end of the double-blind period, in the full-dose combination therapy group, the mean DAS28 (95% CI) in patients who never lost remission in the prior period was 1.7 (1.5, 1.8) compared with 2.7 (2.5, 2.9) in patients who lost remission at one or more time points in the period.

### Predictors of loss of remission

As shown in Table 3 (and summarized in Table 2), several factors—including failure to achieve sustained remission in the open-label period and DAS28 level at weeks 36 and 40—were significant predictors of the loss of DAS28 remission at a single time point in the double-blind period in each treatment group. In addition, the components of DAS28 (e.g., CRP, ESR, TJC, and SJC), patient-reported outcomes (PROs) (e.g., HAQ, PGA, PtGA, and general health and pain scores), and SDAI and CDAI levels were significant predictors across most or all treatment groups but only at week 40. For predictive factors with odds ratios >1, higher values (i.e., greater worsening) were associated with greater likelihood of remission loss. Change from baseline from week 36 to week 40 for these predictors was also significant, with change in PROs being slightly weaker predictors than the best predictors (lack of sustained DAS28 remission, continuous week-36 and week-40 DAS28, and change in DA28 from week 36 to week 40). These predictors were significant even after adjustment for week-36 DAS28, indicating that they explain additional variance beyond what is explained by week-36 DAS28. Similar results were generally found in analyses of predictors of the loss of DAS28 remission plus a change in DAS28 ≥ 0.6 in the double-blind period, as well as loss of SDAI and CDAI remission. However, failure to achieve sustained SDAI or CDAI remission was significantly predictive of the respective SDAI or CDAI loss of remission only in the placebo group, but not in either of the combination therapy groups; change in HAQ from week 36 to week 40 appeared to be one of the strongest predictors of both endpoints.

**Table 3:** Predictors of loss of remission in all treatment groups in the double-blind period

Predictor^a^	Adjusted hazard ratio^b^ (95% CI)		
	ETN 50 mg + MTX *n* = 74	ETN 25 mg + MTX *n* = 56	MTX *n* = 66
Loss of DAS28 remission at single time point in double-blind period			
Lack of sustained DAS28 remission	2.32 (1.44, 3.73)	2.59 (1.48, 4.55)	1.85 (1.24, 2.77)
Sustained weighted DAS28 remission	0.67 (0.56, 0.80)	0.71 (0.59, 0.86)	0.79 (0.68, 0.92)
DAS28 at week 36	2.44 (1.59, 3.73)	2.81 (1.70, 4.63)	1.74 (1.20, 2.51)
DAS28 at week 40	3.24 (2.48, 4.23)	5.88 (4.05, 8.53)	2.26 (1.88, 2.71)
Change in DAS28 from week 36 to 40	3.24 (2.48, 4.23)	5.88 (4.05, 8.53)	2.26 (1.88, 2.71)
ESR at week 40	1.05 (1.02, 1.08)	1.05 (1.02, 1.07)	1.02 (1.01, 1.03)
Change in ESR from week 36 to 40	1.09 (1.06, 1.12)	1.07 (1.04, 1.10)	1.02 (1.01, 1.03)
Sustained weighted SDAI remission	*0.81 (0.58, 1.11)*	0.49 (0.30, 0.78)	*0.80 (0.60, 1.08)*
SDAI at week 40	1.12 (1.08, 1.16)	1.24 (1.17, 1.32)	1.08 (1.06, 1.11)
Change in SDAI from week 36 to 40	1.13 (1.09, 1.18)	1.23 (1.16, 1.30)	1.09 (1.06, 1.12)
Sustained weighted CDAI remission	*0.87 (0.65, 1.17)*	0.49 (0.32, 0.75)	*0.85 (0.64, 1.13)*
CDAI at week 40	1.12 (1.08, 1.16)	1.24 (1.17, 1.31)	1.09 (1.06, 1.12)
Change in CDAI from week 36 to 40	1.13 (1.09, 1.18)	1.23 (1.16, 1.30)	1.09 (1.07, 1.12)
PGA at week 40	1.50 (1.23, 1.81)	1.73 (1.40, 2.12)	1.26 (1.15, 1.39)
Change in PGA from week 36 to 40	1.52 (1.23, 1.88)	1.42 (1.10, 1.83)	1.25 (1.13, 1.38)
PtGA at week 40	1.27 (1.14, 1.42)	1.26 (1.11, 1.43)	1.25 (1.15, 1.36)
Change in PtGA from week 36 to 40	1.28 (1.14, 1.45)	1.49 (1.26, 1.78)	1.28 (1.17, 1.39)
General health VAS at week 40	1.03 (1.02, 1.04)	1.02 (1.01, 1.03)	1.02 (1.02, 1.03)
Change in general health VAS from week 36 to 40	1.04 (1.02, 1.05)	1.03 (1.02, 1.05)	1.03 (1.02, 1.04)
HAQ score at week 40	2.00 (1.31, 3.06)	*1.50 (0.94, 2.38)*	2.04 (1.47, 2.83)
Change in HAQ score from week 36 to 40	2.16 (1.11, 4.20)	3.40 (1.75, 6.59)	3.41 (2.12, 5.46)
TJC at week 40	1.15 (1.09, 1.22)	1.46 (1.31, 1.63)	1.22 (1.14, 1.30)
Change in TJC from week 36 to 40	1.15 (1.08, 1.22)	1.55 (1.37, 1.76)	1.24 (1.16, 1.32)
SJC at week 40	1.11 (0.98, 1.26)	1.44 (1.26, 1.65)	1.18 (1.09, 1.27)
Change in SJC from week 36 to 40	1.31 (1.14, 1.50)	1.40 (1.19, 1.64)	1.23 (1.13, 1.35)
Pain VAS at week 40	1.02 (1.01, 1.03)	1.03 (1.01, 1.04)	1.02 (1.01, 1.03)
Change in pain VAS from week 36 to 40	1.03 (1.01, 1.04)	1.05 (1.04, 1.07)	1.03 (1.02, 1.03)
Loss of DAS28 remission and change in DAS28 ≥ 0.6 in double-blind period			
Lack of sustained DAS28 remission	2.82 (1.64, 4.85)	2.59 (1.44, 4.66)	1.98 (1.33, 2.96)
Sustained weighted DAS28 remission	0.68 (0.57, 0.82)	0.68 (0.55, 0.83)	0.78 (0.67, 0.91)
DAS28 at week 36	*1.21 (0.80, 1.83)*	2.04 (1.25, 3.34)	*1.35 (0.95, 1.90)*
DAS28 at week 40	3.55 (2.67, 4.71)	7.18 (4.79, 10.75)	2.65 (2.17, 3.23)
Change in DAS28 from week 36 to 40	3.55 (2.67, 4.71)	7.18 (4.79, 10.75)	2.65 (2.17, 3.23)
ESR at week 40	1.05 (1.02, 1.08)	1.05 (1.02, 1.07)	1.02 (1.01, 1.03)
Change in ESR from week 36 to 40	1.09 (1.05, 1.12)	1.07 (1.04, 1.10)	1.02 (1.01, 1.03)
Sustained weighted SDAI remission	*0.71 (0.49, 1.01)*	0.47 (0.30, 0.76)	*0.81 (0.60, 1.09)*
SDAI at week 40	1.13 (1.09, 1.18)	1.29 (1.21, 1.38)	1.10 (1.07, 1.12)
Change in SDAI from week 36 to 40	1.15 (1.10, 1.20)	1.26 (1.19, 1.34)	1.11 (1.08, 1.14)
Sustained weighted CDAI remission	*0.80 (0.58, 1.10)*	0.48 (0.31, 0.74)	*0.85 (0.64, 1.14)*
CDAI at week 40	1.13 (1.09, 1.18)	1.28 (1.21, 1.36)	1.10 (1.07, 1.13)
Change in CDAI from week 36 to 40	1.14 (1.10, 1.19)	1.26 (1.19, 1.34)	1.11 (1.08, 1.14)
HAQ at week 40	1.74 (1.08, 2.80)	1.85 (1.15, 2.98)	2.17 (1.55, 3.04)
Change in HAQ from week 36 to 40	*1.74 (0.85, 3.56)*	4.21 (2.11, 8.39)	4.24 (2.61, 6.89)
PGA at week 40	1.55 (1.26, 1.91)	1.92 (1.54, 2.38)	1.30 (1.18, 1.43)
Change in PGA from week 36 to 40	1.70 (1.36, 2.11)	1.60 (1.23, 2.08)	1.30 (1.17, 1.43)
PtGA at week 40	1.25 (1.12, 1.40)	1.34 (1.17, 1.53)	1.30 (1.20, 1.41)
Change in PtGA from week 36 to 40	1.27 (1.12, 1.43)	1.52 (1.27 1.82)	1.35 (1.23, 1.47)
General health at week 40	1.03 (1.01, 1.04)	1.03 (1.01, 1.04)	1.03 (1.01, 1.04)
Change in general health from week 36 to 40	1.04 (1.02, 1.06)	1.03 (1.02, 1.05)	1.03 (1.02, 1.04)
TJC at week 40	1.16 (1.10, 1.23)	1.50 (1.34, 1.69)	1.25 (1.17, 1.34)
Change in TJC from week 36 to 40	1.16 (1.10, 1.23)	1.59 (1.39, 1.80)	1.28 (1.20, 1.37)
SJC at week 40	1.18 (1.03, 1.34)	1.51 (1.32, 1.73)	1.17 (1.08, 1.26)
Change in SJC from week 36 to 40	1.35 (1.16, 1.56)	1.49 (1.27, 1.75)	1.28 (1.17, 1.40)
Pain VAS at week 40	1.02 (1.01, 1.03)	1.03 (1.02, 1.05)	1.02 (1.01, 1.03)
Change in pain VAS from week 36 to 40	1.03 (1.01, 1.04)	1.05 (1.03, 1.07)	1.03, (1.02, 1.04)
	ETN 50 mg + MTX *n* = 72	ETN 25 mg + MTX *n* = 60	MTX *n* = 53
Loss of SDAI remission at single time point in double-blind period			
Lack of sustained SDAI remission	*2.57 (0.90, 7.32)*	*1.86 (0.55, 6.24)*	2.92 (1.08, 7.94)
Sustained weighted SDAI remission	0.67 (0.49, 0.90)	0.62 (0.45, 0.86)	0.68 (0.51, 0.90)
SDAI at week 36	1.56 (1.06, 2.29)	2.00 (1.34, 3.00)	*1.14 (0.80, 1.62)*
SDAI at week 40	1.13 (1.07, 1.19)	1.18 (1.09, 1.29)	1.05 (1.02, 1.08)
Change in SDAI from week 36 to 40	1.13 (1.07, 1.19)	1.18 (1.09, 1.29)	1.05 (1.02, 1.08)
Sustained weighted CDAI remission	0.71 (0.53, 0.95)	0.61 (0.44, 0.83)	0.71 (0.54, 0.92)
CDAI at week 40	1.12 (1.07, 1.18)	1.18 (1.09, 1.29)	1.05 (1.02, 1.08)
Change in CDAI from week 36 to 40	1.12 (1.07, 1.18)	1.19 (1.09, 1.29)	1.05 (1.02, 1.08)
Sustained weighted DAS28 remission	*0.81 (0.63, 1.03)*	*0.93 (0.71, 1.22)*	*0.86 (0.67, 1.10)*
DAS28 at week 40	1.76 (1.20, 2.59)	1.70 (1.05, 2.77)	1.46 (1.19, 1.79)
Change in DAS28 from week 36 to 40	2.09 (1.47, 2.96)	2.55 (1.52, 4.28)	1.62 (1.24, 2.11)
HAQ score at week 40	*1.76 (0.90, 3.44)*	*1.92 (0.86, 4.26)*	2.22 (1.17, 4.21)
Change in HAQ score from week 36 to 40	*2.66 (0.89, 7.96)*	6.13 (1.69, 22.26)	4.25 (1.83, 9.91)
PGA at week 40	1.77 (1.37, 2.29)	1.79 (1.32, 2.42)	1.27 (1.10, 1.47)
Change in PGA from week 36 to 40	1.69 (1.33, 2.14)	1.88 (1.30, 2.70)	1.26 (1.09, 1.46)
PtGA at week 40	1.37 (1.17, 1.61)	1.72 (1.32, 2.24)	1.30 (1.13, 1.50)
Change in PtGA from week 36 to 40	1.40 (1.20, 1.64)	1.73 (1.32, 2.27)	1.30 (1.14, 1.50)
General health VAS at week 40	1.04 (1.01, 1.07)	1.04 (1.01, 1.06)	1.02 (1.01, 1.04)
Change in general health VAS from week 36 to 40	1.05 (1.02, 1.07)	1.04, (1.01, 1.06)	1.03 (1.01, 1.04)
TJC at week 40	1.31 (1.10, 1.56)	1.36 (1.11, 1.68)	*1.05 (1.00, 1.11)*
Change in TJC from week 36 to 40	1.38 (1.14, 1.67)	1.38 (1.13, 1.69)	*1.06 (1.00, 1.12)*
SJC at week 40	1.31 (1.05, 1.63)	*1.15 (0.91, 1.44)*	1.17 (1.01, 1.36)
Change in SJC from week 36 to 40	1.30 (1.04, 1.63)	*1.15 (0.93, 1.44)*	1.17 (1.01, 1.35)
Pain VAS at week 40	1.03 (1.01, 1.05)	1.05 (1.02, 1.08)	1.03 (1.01, 1.04)
Change in pain VAS from week 36 to 40	1.04 (1.02, 1.06)	1.05 (1.03, 1.09)	1.03 (1.01, 1.05)
Loss of CDAI remission at single time point in double-blind period			
Lack of sustained CDAI remission	*2.10 (0.82, 5.40)*	*2.82 (0.83, 9.55)*	2.60 (1.04, 6.48)
Sustained weighted CDAI remission	0.69 (0.51, 0.92)	0.57 (0.42, 0.78)	0.71 (0.55, 0.93)
CDAI at week 36	1.49 (1.04, 2.15)	1.65 (1.14, 2.39)	*1.07 (0.76, 1.51)*
CDAI at week 40	1.15 (1.09, 1.21)	1.21 (1.11, 1.31)	1.05 (1.02, 1.09)
Change in CDAI from week 36 to 40	1.15 (1.09, 1.21)	1.21 (1.11, 1.31)	1.05 (1.02, 1.09)
Sustained weighted SDAI remission	0.63 (0.46, 0.87)	0.59 (0.42, 0.81)	(0.68 (0.51, 0.90)
Sustained weighted DAS28 remission	*0.78 (0.61, 1.00)*	*0.96 (0.76, 1.22)*	*0.81 (0.63, 1.04)*
DAS28 at week 40	1.95 (1.30, 2.91)	2.12 (1.32, 3.38)	1.51 (1.23, 1.86)
Change in DAS28 from week 36 to 40	2.11 (1.47, 3.01)	2.87 (1.77, 4.66)	1.71 (1.31, 2.22)
HAQ score at week 40	2.10 (1.10, 4.00)	*1.87 (0.85, 4.12)*	2.10 (1.10, 4.02)
Change in HAQ score from week 36 to 40	4.00 (1.38, 11.62)	10.88 (3.35, 35.34)	4.34 (1.86, 10.12)
PGA at week 40	1.93 (1.49, 2.49)	1.90 (1.40, 2.56)	1.30 (1.13, 1.50)
Change in PGA from week 36 to 40	1.83 (1.42, 2.35)	2.07 (1.48, 2.91)	1.30 (1.12, 1.51)
PtGA at week 40	1.45 (1.23, 1.70)	1.82 (1.40, 2.37)	1.32 (1.16, 1.51)
Change in PtGA from week 36 to 40	1.46 (1.24, 1.72)	1.75 (1.35, 2.28)	1.34 (1.17, 1.53)
General health VAS at week 40	1.05 (1.02, 1.08)	1.04 (1.02, 1.06)	1.03 (1.01, 1.04)
Change in general health VAS from week 36 to 40	1.05 (1.03, 1.08)	1.04 (1.02, 1.07)	1.03 (1.01, 1.04)
TJC at week 40	1.41 (1.18, 1.68)	1.41 (1.15, 1.74)	1.06 (1.01, 1.12)
Change in TJC from week 36 to 40	1.50 (1.24, 1.82)	1.45 (1.19, 1.76)	1.07 (1.01, 1.13)
SJC at week 40	1.40 (1.12, 1.74)	*1.19 (0.96, 1.49)*	1.20 (1.04, 1.39)
Change in SJC from week 36 to 40	1.39 (1.10, 1.74)	*1.20 (0.97, 1.49)*	1.20 (1.04, 1.39)
Pain VAS at week 40	1.04 (1.02, 1.06)	1.05 (1.03, 1.08)	1.03 (1.01, 1.04)
Change in pain VAS from week 36 to 40	1.04 (1.02, 1.06)	1.06 (1.03, 1.09)	1.03 (1.02, 1.05)

*CDAI* Clinical Disease Activity Index, *CI* confidence interval, *DAS28* Disease Activity Score based on a 28-joint count, *ESR* erythrocyte sedimentation rate, *ETN* etanercept, *HAQ* health assessment questionnaire, *MTX* methotrexate, *PGA* physician global assessment, *PtGA* patient global assessment, *SDAI* Simplified Disease Activity Index, *SJC* swollen joint count, *TJC* tender joint count, *VAS* visual analog scale

^a^Included factors were significant predictors of remission loss in at least one of the three treatment arms, except lack of sustained SDAI/CDAI remission, SJC at week 40, and change in SJC from week 30 to 40 for SDAI and CDAI loss of remission. Sustained remission predictors are weighted. Hazard ratios that are not statistically significant are reported in italics

^b^From Cox proportional hazards model for loss of response, adjusted for baseline of respective outcome (e.g., baseline DAS28, SDAI, or CDAI). Higher hazard ratios denote a higher likelihood of loss of remission. Results cannot be compared across treatment groups because separate models were used for each

**Fig. 4:**
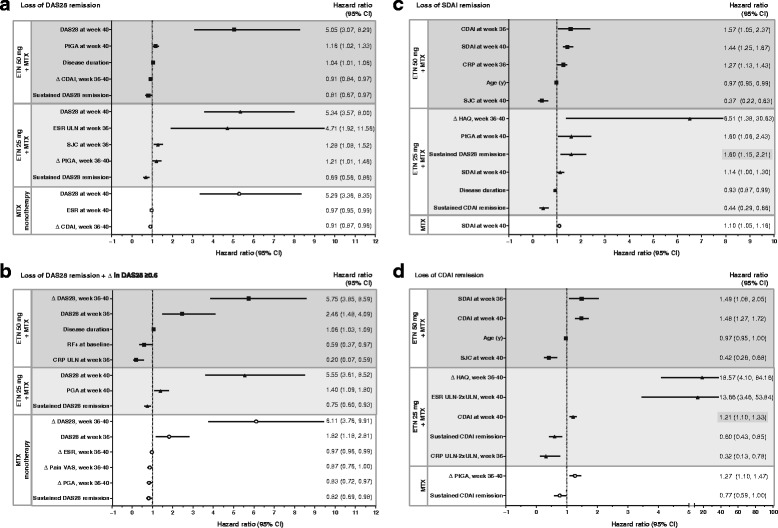
Strongest sets of predictors of loss of Disease Activity Score based on a 28-joint count (DAS28) remission (**a**), DAS28 remission plus change in DAS28 ≥ 0.6 (**b**), Simplified Disease Activity Index (SDAI) remission (**c**), and Clinical Disease Activity Index (CDAI) remission (**d**) in the double-blind period by treatment group using stepwise models. CI, confidence interval; CRP, C-reactive protein; ESR, erythrocyte sedimentation rate; HAQ, health assessment questionnaire; PGA, physician global assessment; PtGA, patient global assessment; RF, rheumatoid factor; SJC, swollen joint count; ULN, upper limit of normal; VAS, visual analog scale

Based on a stepwise model, the strongest set of predictors of the loss of DAS28 remission in the double-blind period in patients maintained on full-dose combination therapy included higher DAS28 and PtGA at week 40; longer disease duration at baseline; less change from baseline in CDAI from week 36 to 40; and lack of sustained DAS28 remission in the open-label period (Fig. 4). In patients who received reduced-dose combination therapy, the strongest set of predictors of loss of DAS28 remission also included higher DAS28 at week 40 and prior lack of sustained DAS28 remission, in addition to ESR levels higher than the ULN at week 36, high SJC at week 36, and greater change in PtGA from weeks 36 to 40. The strongest set of predictors in patients who received methotrexate monotherapy in the double-blind period also included DAS28 at week 40, low ESR levels at week 40, and less change in CDAI from weeks 36 to 40. For loss of SDAI and CDAI remission, the strongest sets of predictors in patients maintained on full-dose combination therapy were similar, including high SDAI/CDAI at week 36 or 40, younger age at baseline, and lower SJC at week 40; however, less similarity was observed in the sets of predictors in the reduced-dose combination and methotrexate monotherapy groups (Fig. 4).

**Fig. 5:**
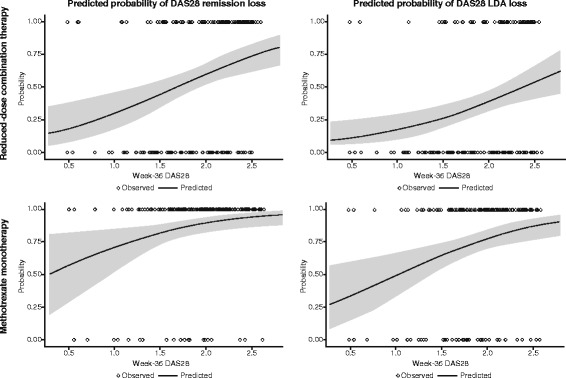
Predicted probability (95% CI) of the loss of Disease Activity Score based on a 28-joint count (DAS28) remission and low disease activity (LDA) in patients receiving reduced-dose combination therapy or methotrexate monotherapy in the double-blind period based on mean DAS28 at week 36 of the open-label period, using logistic regression models. Circles at the top, patients who lost response; circles at the bottom, patients who did not lose response; smooth line, model-predicted probability of loss of response as a function of week-36 DAS28; shadowed area, 95% CI

In analyses using logistic regression models conducted to determine which patients were most likely to experience sustained remission after tapering the etanercept dose or withdrawing the biologic agent, DAS28 after full-dose induction therapy was shown to predict the probability of subsequent loss of response. Patients receiving reduced-dose combination therapy or methotrexate monotherapy who had lower DAS28 at week 36 were less likely to lose remission or LDA in the double-blind period (Fig. 5). Although the relationship between week-36 DAS28 and loss of response in these treatment groups was strong, 95% CIs indicate that the prediction is not precise and no clear cut-point values predictive of loss of remission can be derived. The relationships between week-36 SDAI and CDAI and loss of remission/LDA were less consistent than those for DAS28 (Additional file 3: Figure S2).

**Fig. 6:**
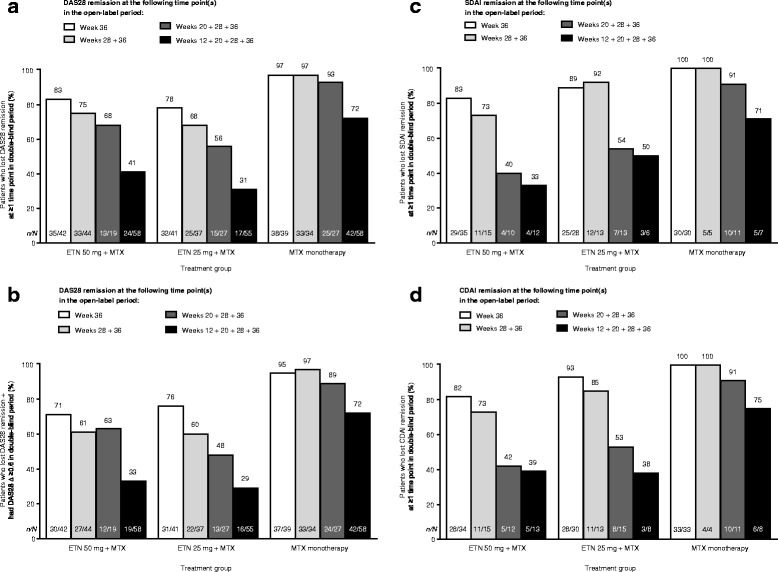
Proportions of patients who lost Disease Activity Score based on a 28-joint count (DAS28) remission (**a**), DAS28 remission and had a change in DAS28 ≥ 0.6 (**b**), Simplified Disease Activity Index (SDAI) remission (**c**), and Clinical Disease Activity Index (CDAI) remission (**d**) at least once at any time point in the double-blind period by duration of remission in the open-label period. *n/N*, numbers of patients who lost remission in the double-blind period/numbers of patients who had remission at specified time points in the open-label period. Patients included in the loss of remission models (i.e., patients with remission at week 36). ETN, etanercept; MTX, methotrexate

Additional analyses showed that in all treatment groups, patients who achieved DAS28 remission only at week 36 or only at weeks 28 and 36 vs. weeks 12, 20, 28, and 36 were much more likely to lose remission at at least one time point after week 36 through week 88, regardless of the index used to measure disease activity (Fig. 6a–d). The trend was less prominent in the treatment group receiving methotrexate monotherapy during the latter period.

Mean DAS28 levels were markedly higher among patients who lost remission at at least one time point than among those who never lost remission in all three treatment groups (Additional file 4: Figure S3). The difference in mean DAS28 between patients who did and did not lose remission was evident at week 36; at this time point, patients who lost remission had a mean DAS28 of approximately 2.2 and patients who never lost remission had a mean DAS28 of 1.6–1.7, which remained at this level even after reduction or withdrawal of etanercept. The difference in DAS28 levels between those who lost and never lost remission was greatest in the methotrexate monotherapy group.

## Discussion

The PRESERVE trial was designed before the development of ACR/EULAR remission criteria and, therefore, DAS28 < 2.6 had been pre-specified as remission in accordance with the trends at that time. However, evidence suggests that patients in DAS28 remission may have substantial residual disease activity [13–15], which is further supported by the present analyses of findings from the PRESERVE trial, as the likelihood of maintaining a good outcome increased with decreasing DAS28 in the “remission” state. The findings of the PRESERVE trial presented in the current report reveal that a large proportion of patients with moderate RA were able to achieve DAS28 remission with full-dose combination etanercept-plus-methotrexate therapy by the end of the open-label period; however, withdrawal of etanercept after achievement of response resulted in a loss of remission in many patients in the double-blind period [6]. As derived from both the univariate and stepwise logistic regression models, patients with moderately active RA who had received full-dose etanercept and methotrexate as induction therapy were significantly more likely to achieve DAS28, SDAI, and CDAI remission if they had lower DAS28/CDAI/SDAI, younger age, BMI <30 kg/m^2^, and lower HAQ score at baseline.

In a subpopulation of patients who were in remission at week 36, DAS28 response was found to be decreased in all treatment groups at the first post-randomization visit (week 40), possibly due to an artificially increased response at week 36 because achievement of DAS28 LDA was required for enrollment in the double-blind period and/or to the changeover from an open-label to a blinded, randomized study. Approximately 25–34% of patients in the full-dose or reduced-dose etanercept-plus-methotrexate groups were not in DAS28 remission at any visit in the double-blind period, having regressed toward their non-remission rate of approximately 25% at week 28 in the open-label period. Interestingly, the mean DAS28 (2.7 (2.5, 2.9)) in patients in the full-dose combination therapy group who lost remission at least once in the double-blind period suggests that patients who lost DAS28 remission at any time point in this period were, on average, near remission at week 88. In contrast, in the methotrexate monotherapy group, the mean DAS28 (3.7 (3.5, 3.9)) in patients who lost remission at least once in the double-blind period indicates a higher level of disease activity that did not similarly approach remission. Most patients who lost DAS28 remission in the double-blind period did so in the month between week 36 (randomization) and week 40; the greatest change in DAS28 scores among patients who lost remission in that period was also observed in this interval. These observations help explain the numerous significant predictors of remission loss identified at week 40 across remission endpoints.

Our findings suggest that patients who did not achieve sustained remission in the open-label period and had higher DAS28 at week 36 were more likely to lose remission with maintenance therapy in the double-blind period. Not surprisingly, patients who achieved remission at only week 36, those who sustained remission at only weeks 28 and 36, and those who sustained remission at only weeks 20, 28, and 36, were at higher risk for loss of remission than patients who sustained remission from week 12 to week 36, indicating that depth of disease control is an important predictor of remission loss. In line with the residual disease activity in patients with a DAS28 < 2.6 mentioned above, the lower the DAS28, and thus the lower the residual disease activity level, the greater the likelihood of sustained response. This result underscores and reinforces the current treat-to-target approach and the importance of adjusting treatment in patients who are not achieving the lowest levels of disease activity currently recommended in ACR/EULAR treatment guidelines [16, 17], as it suggests that the depth and duration of response are relevant. Predictors identified for the maintenance of SDAI and CDAI remission (with the exception of failure to achieve sustained SDAI or CDAI) further support the conclusion of a better outcome with lower disease activity and maintenance of a good response at the time of withdrawal.

Several limitations of the PRESERVE trial and these post hoc analyses should be considered. The study design and endpoints were contemporary at the time at which the study was initiated, and the study excluded patients with mild or severe disease activity and patients with certain comorbid diseases. Physicians could not modify the medication regimens at will (i.e., the regimens were specified by the protocol); patients who left the study were not followed and therefore not assessed. The analyses were limited to the assessments used in the PRESERVE trial and the predictors of remission analyses were limited to data collected in the first 36-week period of the study; in addition, patient follow up occurred at a fixed interval. Sample sizes were small in the methotrexate monotherapy group because most patients lost remission in that group, which resulted in less robust statistical analyses. Moreover, because SDAI and CDAI remission are more stringent endpoints, with fewer patients achieving them than patients achieving DAS28 remission, analyses of the loss of SDAI and CDAI remission had less power to detect differences than analyses of the loss of DAS28 remission. In addition, as noted previously, HRs for predictors of the loss of remission (Table 3) should not be compared across treatment groups because each treatment group has a different rate of loss of remission. Although similar proportions of patients treated with etanercept 50 mg and etanercept 25 mg maintained remission in the double-blind period, differences in HRs for some predictors of the loss of remission were seen between these groups (e.g., change in DAS28 at week 40, change in DAS28 from week 36 to 40).

## Conclusion

The findings of the analyses in the current report suggest that targeting sustained and stringently defined clinical remission in patients receiving full-dose combination etanercept-plus-methotrexate therapy before considering dose or regimen changes may help improve the likelihood that patients will remain in clinical remission 1 year after the changes are made. Patients who are younger and have BMI <30 kg/m^2^, lower HAQ scores, and lower disease activity, as measured by DAS28, SDAI, and CDAI, at baseline may be most likely to achieve a remission state with full-dose combination etanercept-methotrexate induction therapy, whereas those who achieve an early, strong, and durable response to induction therapy are most likely to experience a sustained response after biologic tapering or withdrawal.

## Additional files

Additional file 1: Appendix 1 and Appendix 2. (DOCX 22 kb)

Additional file 2: Figure S1. Summary of patient disposition in the open-label and double-blind periods. *CDAI* Clinical Disease Activity Index, *DAS28* Disease Activity Score based on a 28-joint count, *ETN* etanercept, *LDA* low disease activity, *MTX* methotrexate, *QW* once weekly, *SDAI* Simplified Disease Activity Index. (EPS 1688 kb)

Additional file 3: Figure S2. Predicted probability (95% CI) of the loss of SDAI remission and LDA (**a**) and the loss of CDAI remission and LDA (b) in patients receiving reduced-dose combination therapy or methotrexate monotherapy in the double-blind period based on mean SDAI/CDAI at week 36 of the open-label period, using logistic regression models. Circles at the top indicate patients who lost response; circles at bottom indicate patients who did not lose response; smooth line, model-predicted probability of loss of response as a function of week-36 DAS28; shadowed area, 95% CI. (EPS 2683 kb)

Additional file 4: Figure S3. Mean DAS28 levels in patients who lost remission at ≥1 time point and who never lost remission in the double-blind period. mITT population in double-blind period who had remission at week 36. *CI* confidence interval, *DAS28* Disease Activity Score based on a 28-joint count, *ETN* etanercept, *mITT* modified intent-to-treat, *MTX* methotrexate. (EPS 1406 kb)

## Abbreviations

ACPA: Anti-citrullinated peptide antibody
ACR/EULAR: American College of Rheumatology/European League Against Rheumatism
BMI: Body mass index
CDAI: Clinical Disease Activity Index
CI: Confidence interval
CRP: C-reactive protein
DAS28: Disease Activity Score in 28 joints
ESR: Erythrocyte sedimentation rate
ETN: Etanercept
HAQ: Health Assessment Questionnaire
JSN: Joint space narrowing
LDA: Low disease activity
mITT: Modified intent-to-treat
mTSS: Modified total Sharp score
MTX: Methotrexate
PGA: Physician global assessment
PRO: Patient-reported outcome
PtGA: Patient global assessment
QW: Once weekly
RA: Rheumatoid arthritis
RF: Rheumatoid factor
SD: Standard deviation
SDAI: Simplified Disease Activity Index
SJC: Swollen joint count
TJC: Tender joint count
TNF: Tumor necrosis factor
ULN: Upper limit of normal
