# Supplementary material for: Predictors of remission with etanercept-methotrexate induction therapy and loss of remission with etanercept maintenance, reduction, or withdrawal in moderately active rheumatoid arthritis: results of the PRESERVE trial
Source: Arthritis Res Ther. 2018 Jan 16;20:8. doi: 10.1186/s13075-017-1484-9 (PMC5771183; doi:10.1186/s13075-017-1484-9)
Supplement: Supplementary file 1 — Appendix 1 and Appendix 2. (DOCX 22 kb) [file 13075_2017_1484_MOESM1_ESM.docx]

**Additional file 1**

**Supplemental Appendix 1**

**PRESERVE Post Hoc Statistical Analysis Plan 1**

# OBJECTIVES / RATIONALE

- Objective: This plan outlines the specifications and the procedures needed to analyze baseline disease characteristics that can predict DAS28, CDAI, and SDAI remission.
- Rationale: Much interest has been generated regarding predictors of DAS28 remission. This analysis will look at baseline disease characteristics (including DAS28/SDAI/CDAI components) and patients’ early response to etanercept in order to explore which factors may be predictive of remission. When the second period of PRESERVE is completed, an investigation of factors that predict a sustained response among period-one responders may also be considered.

# METHODOLOGY

# Observed case (OC) analysis to be conducted of changes from baseline to 36 weeks.

#

# POPULATION

- All patients in the modified intention-to-treat (mITT) population of Period 1 of PRESERVE who had baseline and week-36 data.
- Patients who had week-36 DAS28/SDAI/CDAI values will be included in the respective analyses of DAS28/SDAI/CDAI remission.

# ENDPOINTS/ MEASUREMENTS

## Primary endpoints

## Week 36 DAS28 remission

## Week 36 SDAI remission

## Week 36 CDAI remission

## Baseline predictors

- Baseline characteristics (analyzed as continuous and/or dichotomous predictors)
- Age >40 vs. ≤40, >40–≤65 vs. ≤40, and ≥65 vs. <65
- BMI
- BMI 18.5–25 vs. <18.5 kg/m^2^, 25–30 vs. <18.5 kg/m^2^, ≥30 vs. <18.5 kg/m^2^
- Gender
- Prior/current smoker
- Disease duration
- Disease duration: ≤6 vs. >24 months, >6–≤12 vs. ≤6 months, >12–≤24 vs. ≤6 months
- Anti-citrullinated peptide antibody (ACPA) pos vs. neg
- Rheumatoid factor (RF) pos vs. neg
- CRP
- CRP > vs. ≤ upper limit of normal (ULN)
- ESR
- ESR > vs. ≤ ULN
- DAS28
- DAS28: 4.1–4.4 vs. ≤4.1, 4.4–4.7 vs. ≤4.1, and >4.7 vs. ≤4.1
- SDAI
- CDAI
- CDAI, 14.5–17 vs. ≤14.5, 17–20 vs. ≤14.5, and >20 vs. ≤14.5
- SDAI
- SDAI, 15.5–18.4 vs. ≤15.5, 18.4–21.8 vs. ≤15.5, and >21.8 vs. ≤15.5
- PGA
- PtGA
- HAQ
- HAQ score, >0.5–≤1.0 vs. ≤0.5, >1.0–≤1.5 vs. ≤0.5, and >1.5 vs. ≤0.5
- mTSS, erosion and joint space narrowing (JSN)

#

# STATISTICAL METHODS

- Primary analysis
  - Remission is defined as DAS28 <2.6, SDAI ≤3.3, or CDAI ≤2.
  - Demographic and baseline disease characteristics will be analyzed as possible predictors of week 36 remission.
  - Univariate logistic regression models will be generated for each baseline predictor (continuous or categorical) listed in the “Baseline predictors” section above.
  - These models will be adjusted for their respective baseline DAS28, SDAI, or CDAI. Odds ratios and 95% CIs will be calculated.
- Secondary analyses
  - Step-wise logistic regression of baseline variables to determine which are most predictive of outcomes.
  - Authors reviewed the list of baseline predictors and selected the most clinically relevant predictor.
  - If predictors were highly correlated (e.g. baseline CRP and baseline ESR), authors selected one of the two.
  - The final list of predictors selected by authors are listed below.

| Age | ≤40 vs. >40 to ≤65 vs. >65 |
| --- | --- |
| BMI | BMI <25 vs. ≥25 to <30 vs. ≥30 |
| CRP | ≤ULN vs. >ULN to ≤3*ULN vs. >ULN |
| Disease duration | ≤6 mon, >6 to ≤12, >12 to ≤24, >24 |
| Gender | Male vs. female |
| Smoker | Prior or current Smoker Y vs. N |
| Baseline mTSS |  |
| HAQ | ≤0.5, >0.5 to ≤1, >1 to ≤1.5, >1.5 |

- Baseline DAS28/CDAI/SDAI were required to be included in the final stepwise model for DAS28/CDAI/SDAI remission.
- Radiographic variables will be included in univariate analyses but not in stepwise analyses because week 36 x-ray data were not available for approximately 65 patients

**Supplemental Appendix 2**

**PRESERVE Post Hoc Statistical Analysis Plan 2**

# OBJECTIVES / RATIONALE

- *Objective*: This plan outlines the specifications and the procedures needed to analyze demographic and disease characteristics from baseline of period 1 and disease characteristics at the end of period 1/baseline of period 2 and during period 2 in order to identify predictors of loss of DAS28 Clinical Remission during period 2.
- *Rationale*: Much has been written about identifying predictors of clinical and radiographic outcomes in patients treated with DMARD therapy. However, the distinctive feature of period 2 of the PRESERVE study is the focus on maintaining the response induced/achieved on full dose etanercept plus methotrexate. Exploration of predictors of loss of response, specifically loss of clinical remission, has the potential to inform clinical decision-making by highlighting demographic and clinical variables that may signify a need to adjust treatment in order to sustain remission.This analysis will look at demographic and disease characteristics from baseline of period 1 and disease characteristics at week 36 (end of period 1/baseline of period 2) and during period 2 in order to explore predictors of loss of clinical remission during period 2. Given the finding that 47% in the MTX-only arm versus only 17% of patients in the full-dose combination therapy arm lost response (DAS28 LDA >3.2) by 12 weeks, an additional analysis will look at predictors of prompt loss of response after 12 weeks in period 2 (week 48 of the study). Recognizing that greater precision for predicting loss of response may depend on examining changes or trends in selected variables more proximate to the actual time of loss of response, an additional analysis will look at potential predictors not only at week 36 but also at potential time-dependent predictors at some of the subsequent visits during period 2 prior to the final visit at week 88 (weeks 40, 48).

# POPULATION

All patients in the mITT population of period 2 who achieved week 36 DAS28 clinical remission (163/201 [81.1%], 160/201 [79.6%], and 158/197 [80.2%] in the ETN 50 + MTX, ETN 25 + MTX, and PBO/MTX arms, respectively) will be included in all period 2 DAS28 remission analyses. All patients in mITT population of period 2 who achieved week 36 CDAI remission will be included in period 2 SDAI and CDAI remission analyses.

# PROTOCOL INCLUSION / RATIONALE

- L-Wyeth 0881A1-4423
- Pfizer B1801003

# ENDPOINTS / MEASUREMENTS

## Primary endpoint: First loss of DAS28 clinical remission (defined as DAS28 ≥2.6) during period 2.

## Secondary endpoints

- First loss of DAS28 clinical remission that is combined with an absolute increase of ≥0.6 in the DAS28 score during period 2.
- First lost of CDAI remission during period 2.
- First lost of SDAI remission during period 2.

## Predictors

- Baseline of period 1 demographic and disease characteristics: age [continuous], gender [nominal], BMI [ordinal], disease duration [continuous], anti-CCP [nominal], RF [nominal]; assumption is that these values do not change or that any change is minimal or applied equally across all subjects, so they will be treated as week 36 variables (meeting the time-independence assumption of Cox proportional hazard modelling).
- At 36 weeks: end of period 1/baseline of period 2 disease characteristics: absolute DAS28 at week 36 from baseline of period 1 [continuous], TJC [continuous, 28 joints], SJC [continuous, 28 joints], ESR [continuous], CRP [continuous], HAQ [continuous], erosion score [continuous], joint space narrowing score [continuous], physician global assessment [continuous], patient global assessment [continuous], general health assessment VAS [continuous], pain assessment VAS [continuous], mTSS, erosion, and JSN.
- Change in the above variables at week 36.
- Week 40 for above variables (except mTSS, erosion, and JSN, which wasn’t collected then).
- Change at week 40 for above variables (except mTSS, erosion, and JSN, which wasn’t collected then).
- Sustained remission during period 1 (i.e. DAS28 <2.6 at weeks 12, 20, 28, and 36) versus non-sustained remission during period 1.
- Sustained remission will also be characterized by degree of sustainability – sustained at weeks 12, 20, 28, and 36 versus sustained at weeks 20, 28, and 36 versus sustained at weeks 28 and 36 versus only at week 36 versus non-sustained.

# STATISTICAL METHODS (as originally submitted)

| Cox proportional hazards modeling will be utilized to determine the relationship between the “hazard”, first loss of DAS28 clinical remission, and a range of potential demographic and clinical covariates.Similar modeling will be utilized for first loss of DAS28 clinical remission that is combined with an absolute increase of ≥0.6 in the DAS28 score, as well as first loss of SDAI remission and first loss of CDAI remission.For each endpoint, separate models will be run for the three arms during period 2 (etanercept 50 mg plus methotrexate, etanercept 25 mg plus methotrexate, placebo plus methotrexate).Each predictor listed above will be analyzed separately in a Cox model. Each DAS28 remission, CDAI remission, and SDAI remission will be adjusted for their baseline DAS28, CDAI, or SDAI. For each predictor, a hazard ratio value and the 95% confidence interval will be generated.A subgroup analysis will be conducted between those patients who never lost DAS28 remission versus those who did, with comparison of mean DAS28 values (and other descriptive statistics) at each time point by treatment arm. |
| --- |

#

# STATISTICAL METHODS (in response to peer review)

- Additional analyses were performed to address the following peer reviewer comment (July 2017): *“...*tell the readers of AR&T who will (likely) benefit from tapering and who will benefit from complete withdrawal <<of biologic>>....”
- Unfortunately, the cut points for week-36 DAS28, CDAI, SDAI, and HAQ that best predicted loss of remission after tapering or withdrawal of biologic combination therapy could not be pinpointed because most patients lost remission for at least one time point in the randomized double-blind period (e.g., 64% of patients in the full-dose combination group, 56% of those in the reduced dose combination group, and 87% of those in the methotrexate monotherapy group lost DAS28 remission at least once). Moreover, small sample sizes (especially in the CDAI/SDAI analysis groups and methotrexate monotherapy group) limited the analyses‘ ability to provide clear and robust guidance on who would maintain remission after tapering or withdrawal of biologic treatment.
- Given the above-mentioned limitations, stepwise models were created for each endpoint and each treatment to determine which subset of predictors were the ‘best’ set of predictors (i.e., demographic and week-36/-40 predictors).
- In addition, the relationship between clinical predictors and the probability of loss of remission was analyzed using the following pairs in univariate logistic regression models: week-36 DAS28 vs loss of DAS28 remission; week-36 DAS28 vs loss of DAS28 remission plus DAS28 change ≥0.6; week-36 SDAI vs loss of SDAI remission; and week-36 CDAI vs loss of CDAI remission.
